# Fistula formation in recurrent sigmoid diverticulitis - a domain of laparoscopic surgery?

**DOI:** 10.1007/s00423-025-03924-0

**Published:** 2025-11-21

**Authors:** Lennart Zimniak, Stephan Gretschel, Hendrik Christian Albrecht, Kjell Sonnenberg, Christoph Wullstein, Attila Dubecz, Michael Karg, Joerg-Peter Ritz

**Affiliations:** 1 Department of General, Visceral, Thoracic and Vascular Surgery, University Hospital Ruppin-Brandenburg, Neuruppin, Germany; 2 Brandenburg Medical School, Neuruppin, Germany; 3 Department of General and Visceral Surgery, HELIOS Klinikum Krefeld, Krefeld, Germany; 4 Department of General and Visceral Surgery, HELIOS Klinikum Erfurt, Erfurt, Germany; 5 Department of General and Visceral Surgery, HELIOS Klinikum Schwerin, University Campus Medical School Hamburg, Schwerin, Germany

**Keywords:** Recurrent sigmoid diverticulitis, Colovesical fistula, Colovaginal fistula, Laparoscopy, Open surgery, Comparative study

## Abstract

**Introduction:**

Chronic sigmoid diverticulitis is the most common benign cause of sigmoid-bladder fistulas (SBF) and sigmoid-vaginal fistulas (SVF). This multicenter retrospective comparative study analyzed the perioperative and postoperative outcomes between laparoscopic and open surgical procedures.

**Methods:**

The study included 101 patients from four German hospitals who underwent elective sigmoid resection for SBF, SVF, or combined fistulas between January 2010 and July 2024. Patient data were retrospectively analyzed, comparing outcomes based on the surgical approach.

**Results:**

Of the 101 patients, 70 (69.3%) had a sigmoid-bladder fistula, 29 (28.7%) had a sigmoid-vaginal fistula, and 2 (2%) had a combined fistula. Fifty-seven patients (56.4%) underwent open surgery, while 44 (43.6%) had laparoscopic surgery. The median hospital stay was significantly shorter in the laparoscopic group (11 days vs. 16 days, *p* = 0.016). The laparoscopic group also showed earlier removal of drains (4 days vs. 5.5 days, *p* = 0.044), shorter intensive care unit (ICU) stays (0.5 days vs. 1.5 days, *p* = 0.026) and earlier return of bowel function (3 days vs. 5 days, *p* < 0.001). No significant differences were observed in anastomotic leakage rates (1 in the laparoscopic group vs. 7 in the open group, *p* = 0.066), mortality rates (1 in the laparoscopic group vs. 4 in the open group, *p* = 0.384), wound infection rates (7 in the laparoscopic group vs. 15 in the open group, *p* = 0.234) and operating time (206 min in the laparoscopic group vs. 159 min in the open group *p* = 0.133).

**Conclusion:**

Laparoscopic procedures, if technical possible, potentially demonstrate superior postoperative outcomes compared to open surgery for the treatment of fistulizing sigmoid diverticulitis in several parameters without increasing risk or operating time.

## Introduction

Colonic diverticulosis and associated diverticulitis are common conditions. Fistulas to the bladder or vagina represent a significant complication of chronic sigmoid diverticulitis. Approximately 90% of fistulas in diverticulitis involve either one of these two organs [1]. The incidence of colovesical or colovaginal fistula formation in chronic sigmoid diverticulitis is reported in the literature to range between 1% and 4% [2, 3]. The primary cause of such fistulas, aside from abscessing inflammation, is the close anatomical proximity between the affected secondary organ and an acutely inflamed or covered-perforated diverticulum. A thorough patient history is the key instrument for diagnosing colovesical fistulas, with pathognomonic symptoms such as complicated, recurrent urinary tract infections, pneumaturia, or fecaluria. In colovaginal fistulas, patients typically present with passage of stool or gas through the vagina and recurrent episodes of vaginitis. This study aims to provide a multicenter comparison of laparoscopic versus open resective approaches in the surgical management of fistulizing sigmoid diverticulitis, a topic for which current literature remains limited.

## Patients and methods

Between January 2010 and July 2024, all patients who underwent elective resective surgical therapy for fistulizing sigmoid diverticulitis were included in this observational study within a network of four German tertiary care hospitals. Patients with a history of malignancy in the urogenital or lower gastrointestinal tract, chronic inflammatory bowel diseases, or prior pelvic radiation therapy were excluded from the study. Diagnosis was made based on clinical symptoms and confirmed by imaging. A contrast-enhanced abdominal CT scan was performed in all patients. In suspected colovesical fistulas, cystoscopy and cystography was frequently added. In suspected colovaginal fistulas, vaginal inspection was commonly performed to support diagnosis. Data were collected from patient records, discharge summaries, and surgical reports, including the surgical approach (laparoscopic/open surgery), operative time, placement of drains, and postoperative course.

**Fig. 1:**
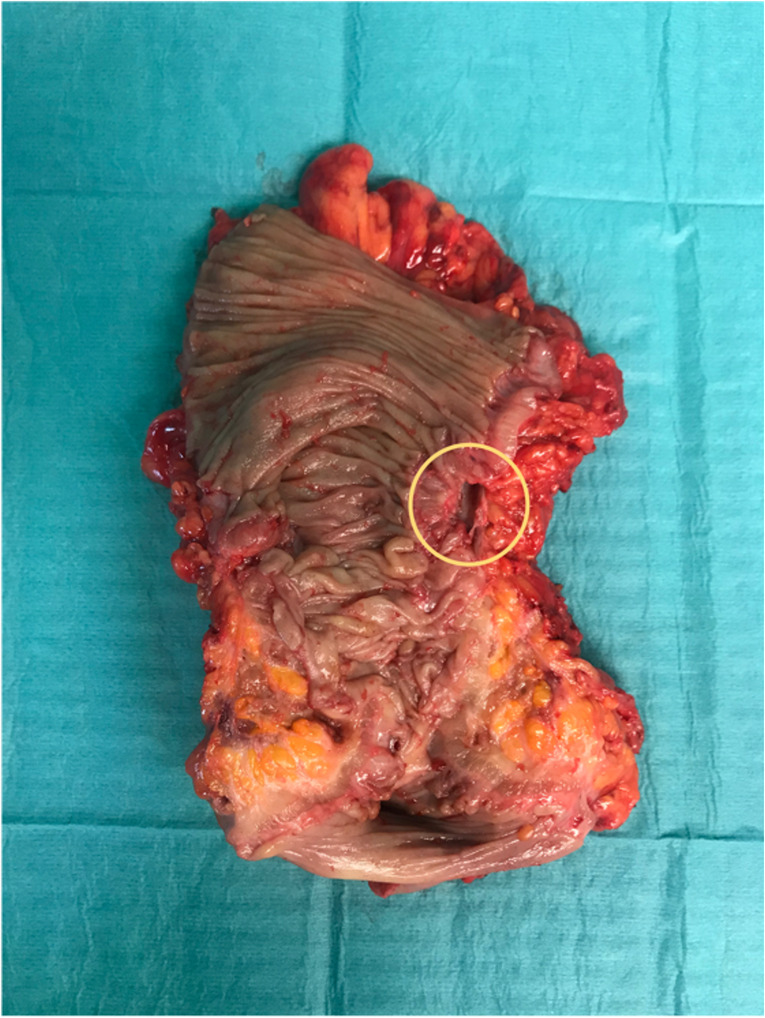
Resection specimen of a sigmoid colon with a clear fistula (yellow marking)

The resective procedure involved segmental colonic resection near the bowel wall (sigmoid resection) (Fig. 1) with resolution of the fistulous connection and repair of the fistula target organ (bladder/vagina) Patients who underwent conversion to an open approach were assigned to the laparoscopic group according to an intention-to-treat analysis. Interprofessional teams were defined as surgical procedures in which, in addition to the visceral surgeon performing the sigmoid resection, a urologist or gynecologist was involved to assist in the repair of the affected target organ.

Metric variables were compared using the Mann-Whitney U test. The Chi-square test and Fisher’s exact test were employed to compare the distribution of categorical variables between independent groups. P-values < 0.05 were considered statistically significant. Statistical analyses were performed using IBM SPSS Statistics Version 29.0.0.0. The study was approved by the Ethics Committee of Rostock University with the registration number A2019-0033.

## Results

**Fig. 2:**
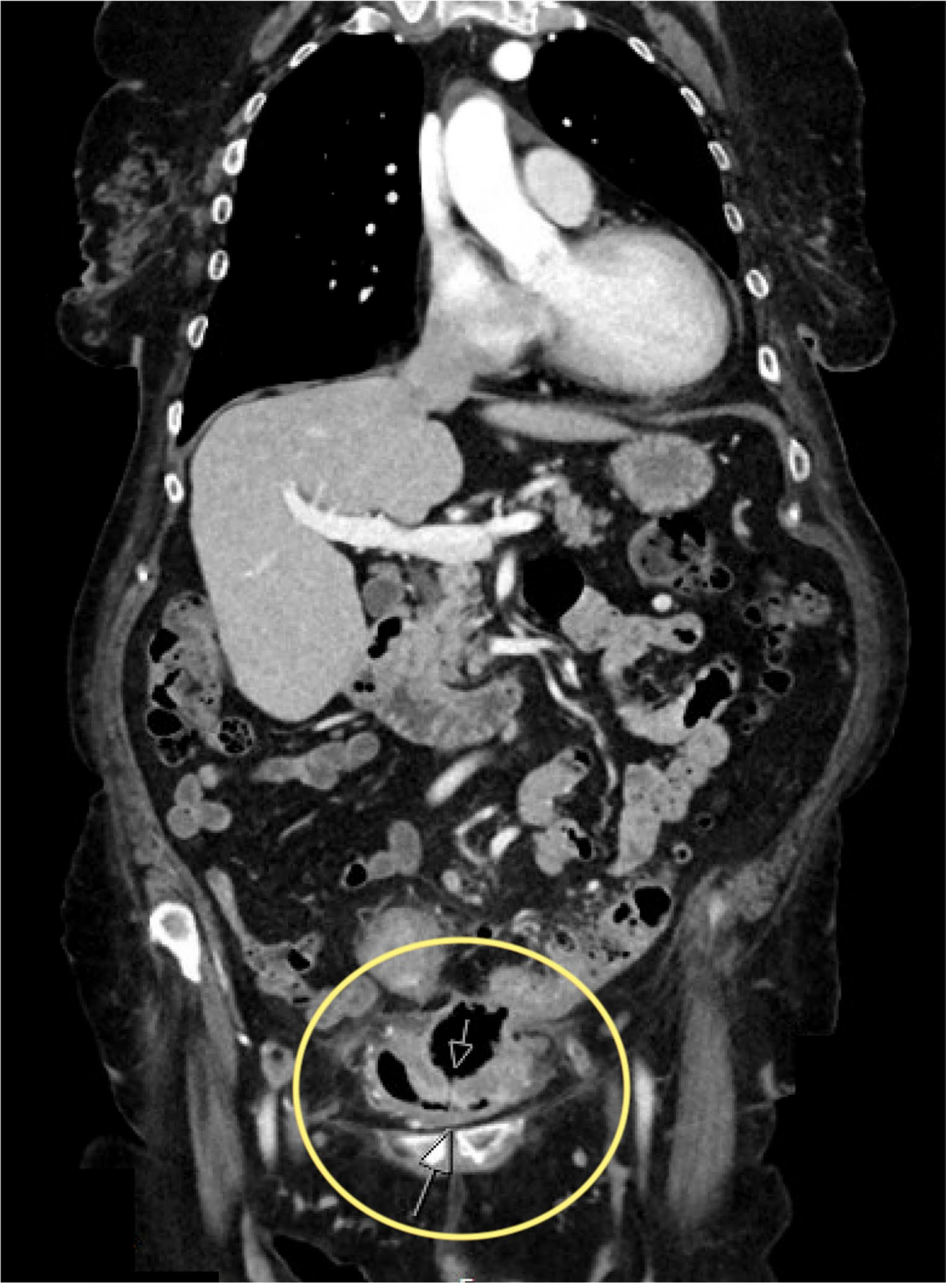
CT Imaging of a sigmoid bladder Fistula (SVF) highlighted by arrow

During the study period, a total of 101 patients underwent surgical treatment for sigmoid diverticulitis with fistula formation. Of these, 70 patients (69.3%) had a sigmoid-bladder fistula (SBF), 29 patients (28.7%) had a sigmoid-vaginal fistula (SVF), and 2 patients (2%) had a combined sigmoid-bladder/sigmoid-vaginal fistula. The cohort consisted of 53 male patients (52.5%) and 48 female patients (47.5%). The median age was 70 years and median BMI was 27.5 kg/m^2^. There were no group differences regarding sex, age or BMI (Fig. 2).

Among the included patients, 57 (56.4%) underwent open surgery, while 44 (43.6%) were treated laparoscopically, whereby 14 cases (31.8%) were converted. The open surgical approach was more frequently chosen when interprofessional teams were involved, with 24 cases in the open group compared to 8 in the laparoscopic group (*p* = 0.017). Intraabdominal drainage was significantly more commonly placed in the open procedure group (*n* = 44) compared to the laparoscopic group (*n* = 12) (*p* < 0.001). The median hospital stay was significantly shorter in the laparoscopic group compared to the open surgery group (11 days, IQR 8–17.5 vs. 16 days, IQR 11.5–23.5; *p* = 0.016). Similarly, the median postoperative ICU stay was reduced in the laparoscopic group (0.5 days, IQR 0–1 vs. 1.5 days, IQR 1–3; *p* = 0.026). The median time to removal of abdominal drains was shorter in the laparoscopic group (4 days, IQR 2–5) compared to the open group (5.5 days, IQR 4–7.25; *p* = 0.044). Laparoscopic surgery also resulted in faster restoration of bowel function (3 days, IQR 2–5 vs. 5 days, IQR 4–7.75; *p* < 0.001). The median operating time was comparable between the two groups without significant differences (206 for laparoscopic vs. 159 min for open surgery; *p* = 0.133).

Postoperative complications included anastomotic leakage in 8 patients (7.9%), wound infections in 22 patients (21.8%), and reoperation in 16 patients (15.8%). The surgical approach (laparoscopic vs. open) did not significantly affect the rates of anastomotic leakage (*p* = 0.066), wound infection (*p* = 0.234), or reoperation (*p* = 0.411). Postoperative mortality was 5% (*n* = 5), affecting exclusively patients with elevated preoperative risk profiles (ASA III–IV), with no significant difference between the laparoscopic and open groups (*p* = 0.384); causes of death were sepsis with subsequent multiorgan failure or severe pneumonia. Prolonged antibiotic therapy was administered to 62 patients (61.4%) and was significantly more common in the open procedure group (*n* = 42) vs. the laparoscopic group (*n* = 19) (*p* = 0.002). Foley catheter placement was required in 95 patients. Table one and two summarizes these results (Tables 1 and 2). Anastomotic leakage and mortality occurred more frequently in patients with ASA III and ASA IV. Both outcomes showed statistically significant associations with ASA classification (*p* = 0.036 and *p* = 0.012, respectively) (Table 3).

**Table 1:** Patient and group characteristics overview

Category	Laparoscopic group (*n*/median)	Open group (*n*/median)	Total (*n*)	*P* value
Number of patients	44	57	101	0.166
Age (years)	58	72	70	0.842
BMI (kg/m²)	27.6	27.3	27.5	0.911
Male	25	28	53	0.845
Female	19	29	48	0.249
Sigma-bladder-fistula (SBF)	29	41	70	0.351
Sigma-vaginal-fistula (SVF)	15	14	29	0.840
Combined-fistula (CF)	0	2	2	**< 0.001**
Preoperative CRP (mg/dl)	58	64	61.1	0.671
Preoperative Leucocytes G/l	11.5	12.7	12.2	0.473
Open procedure	-	57	57	-
Laparoscopic procedure	44	-	44	-
Operating time (min)	206	159	163	0.133
Conversion rate	14	-	14	-
Abdominal pre operations	21	35	56	0.172
Interprofessional teams	8	24	32	**0.017**
Anastomosis perfomed	42	50	92	0.321
Hand sutured Anastomosis	2	14	16	**0.040**
Stapler sutured Anastomosis	38	38	76	0.962
Stoma due to revision	1	1	2	0.933
Transurethral foley catheter	33	45	78	0.641
Suprapubic foley catheter	11	12	23	0.819
Abdominal drainage placement	12	44	56	**< 0.001**

**Table 2:** Comparison between laparoscopic and open procedures regarding peri- and postoperative outcome

Category	Laparoscopic group (*n*/median)	IQR	Open group (*n*/median)	IQR	*P* value
Hospital stay (d)	11	8–17.5	16	11.5–23.5	**0.016**
Foley catheter removal	7	3–9	8	6.5–11.5	0.158
Abdominal drainage removal	4	2–5	5.5	4–7.25	**0.044**
ICU care (d)	0.5	0–1	1.5	1–3	**0.026**
Return of bowel function	3	2–5	5	4–7.75	**< 0.001**
Postoperative antibiotic therapy	19	-	42	-	**0.002**
Mortality	1	-	4	-	0.384
Anastomosis leackage	1	-	7	-	0.066
Wound infection	7	-	15	-	0.234
Relaparotomy	5	-	11	-	0.411
Postoperative bleeding	2	-	2	-	0.988

**Table 3:** Impact of ASA classification (1–2 vs. 3–4) on key surgical outcomes

Outcome	ASA 1	ASA 2	ASA 3	ASA 4	*p*-value
Open approach (n)	0	42	11	4	0.119
Mortality (n)	0	1	3	1	**0.012**
Anastomotic leakage (n)	0	3	4	1	**0.036**
Conversion (n)	0	10	4	0	0.530

## Discussion

Colovesical fistulas were first described by Cripps in 1888 [4]. Sigmoid diverticulitis is estimated to be responsible for two-thirds of cases in patients with sigmoid-bladder fistulas (SBF). Other reported causes include malignancies of the colon or bladder, chronic inflammatory bowel diseases such as Crohn’s disease or ulcerative colitis, and iatrogenic effects like radiation therapy or prior abdominal surgeries [2–5]. Despite the typically pathognomonic symptoms (pneumaturia, complicated and recurrent urinary tract infections, feculent vaginal discharge), the time between symptom onset and diagnosis of sigmoid-bladder or sigmoid-vaginal fistulas is often prolonged. In our study, 96% of patients with SBF had preoperative nitrite-positive urinary tract infections. Preoperative inflammatory markers were elevated, with a median CRP of 61.1 mg/dl and leukocyte count of 12.2 G/l. This suggests that even elective treatment of this condition differs from other planned visceral procedures. Chronic infection may contribute to the high wound infection rates in our cohort. Postoperative antibiotic therapy was administered in 61.4% of patients, reflecting the chronic inflammatory burden and frequent urologic or gynecologic organ repair. In line with ERAS and DGAV recommendations, antibiotic continuation should be individualized based on clinical infection markers rather than routine extension.

Most patients with SBF in this sample were male (75.7%), predisposing them to fistula formation. This observation aligns with other studies identifying male sex as a risk factor for this condition. In a retrospective study on the diagnosis and surgical treatment strategies for SBF in chronic sigmoid diverticulitis, Melchior et al. included only 7 female patients (14%) among a total of 49 cases over a 25-year period [6]. Among the 48 female patients in our study group, 84.6% had a history of hysterectomy, placing them in a high-risk group for fistula formation. This finding underscores the protective effect of the uterus as an anatomical buffer between the sigmoid colon and the bladder or vagina, as previously described in the literature [3–5]. 

**Fig. 3:**
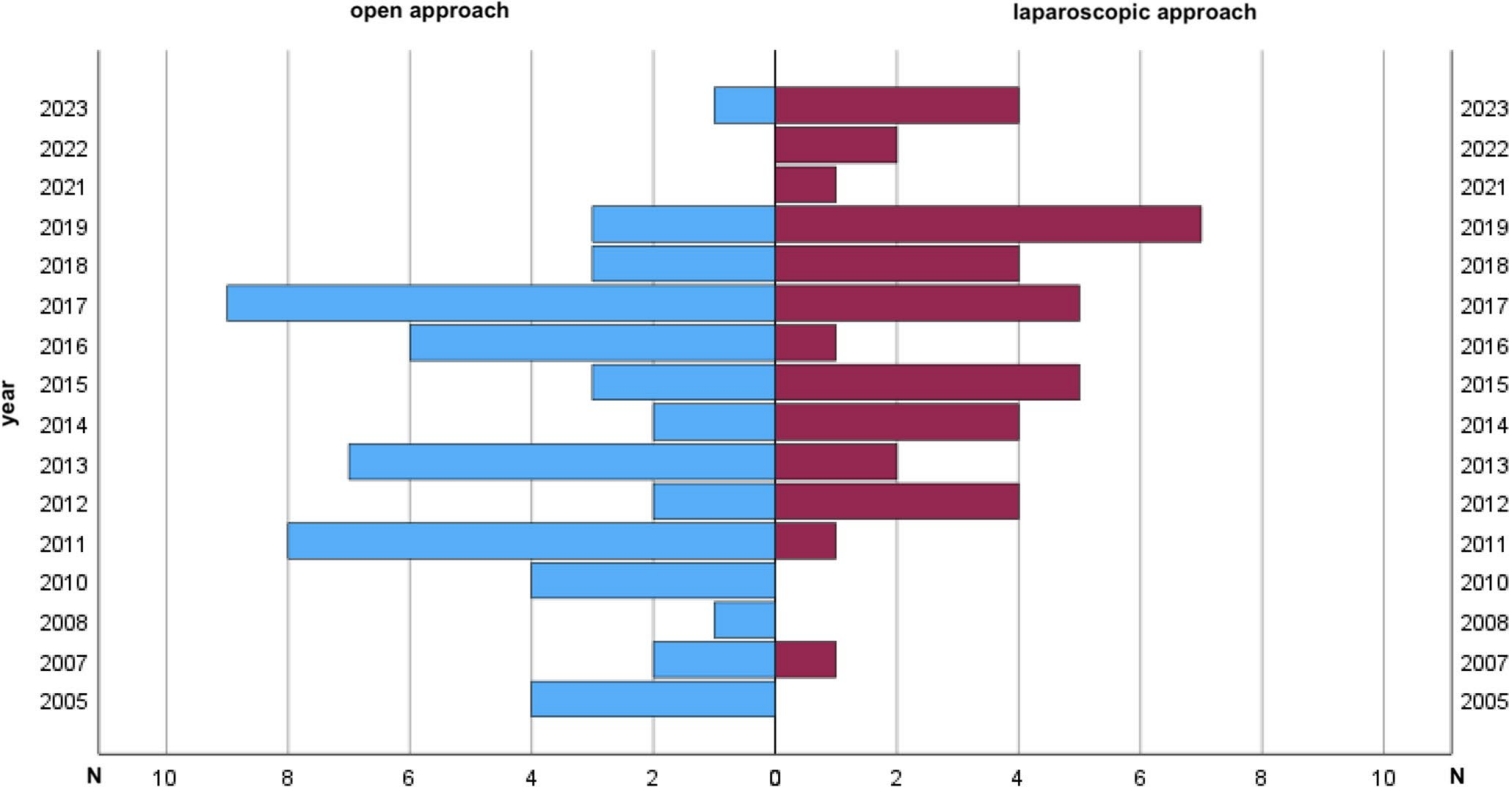
Trends in Surgical Approaches: A Yearly Comparison of Open and Laparoscopic Procedures

The aim of this study was to investigate whether the surgical approach impacts perioperative and postoperative treatment outcomes for these conditions. Over the past 30 years, the field of minimally invasive surgery has rapidly advanced, establishing laparoscopy as a safe and often superior method in visceral surgery [7–17]. This trend is reflected by a marked increase in the proportion of laparoscopic procedures over the study period, particularly after 2015, corresponding with the broader establishment of minimally invasive colorectal surgery in clinical practice. This temporal development is illustrated in Fig. 3. (Figure 3) Anyway, preoperative factors such as prior abdominal or pelvic surgeries and inflammatory changes (e.g., sigmoid diverticulitis) can complicate laparoscopic procedures. For this reason, some surgeons still advocate for an open surgical approach in complex visceral procedures. Diverticulitis-associated fistulas further complicate this already challenging condition [18]. Given this complexity, standardizing and improving current treatment protocols is essential.

The final choice between laparoscopic and open surgery was likely influenced by case complexity rather than baseline patient characteristics. While there were no relevant differences in BMI, age, or inflammatory markers between the groups, patients in the open surgery group had a higher rate of prior abdominal surgeries, which may have contributed to the decision for an open approach due to anticipated adhesions or altered anatomy. Additionally, interprofessional surgical teams were more frequently involved in open procedures, suggesting that cases requiring multidisciplinary expertise were preferentially managed with an open approach. Moreover the higher frequency of intraabdominal drainage placement in the open surgery group may indicate that more complex cases were preferentially treated with an open approach, even though our data did not provide direct evidence for this assumption. These factors highlight the challenges in standardizing treatment selection in this patient population and should be considered when interpreting perioperative outcome comparisons.

In our study, conversion from laparoscopic to open surgery occurred in 14 out of 44 cases (31.8%). The conversion rate aligns with previously reported values for minimally invasive colorectal surgery in complex cases. Importantly, statistical analysis did not reveal any significant association between conversion and key preoperative patient characteristics, including age, BMI, fistula size, CRP, leukocyte count, or symptom duration. This suggests that the decision to convert was likely influenced by intraoperative findings rather than preoperative factors. Possible reasons for conversion may include extensive adhesions, difficult anatomical conditions, or intraoperative complications. A trend towards reduced conversion rates over time, as might be expected with growing experience in laparoscopic colorectal surgery, could not be demonstrated in our cohort. Robotic-assisted approaches may further facilitate minimally invasive management of complex fistulas and therefore represent a promising focus for future research. Prospective studies with detailed intraoperative documentation are needed to better identify factors contributing to conversion and to determine whether robotic systems can reduce conversion rates.

Our findings demonstrated that laparoscopic procedures significantly reduced hospital stays, ICU stays and indwelling abdominal drainage. Furthermore, bowel function returned significantly earlier in the laparoscopic group. Although no prior studies have directly compared surgical approaches for SBF/SVF caused by diverticulitis, it is reasonable to assume that the well-documented advantages of laparoscopy over open surgery apply to this condition as well [18]. This assumption is supported by the findings of Menenakos et al., who reported a mean hospital stay of 10 days in a consecutive case series of 18 patients undergoing laparoscopic sigmoid resection with bladder repair for SBF caused by diverticulitis [19]. 

Other factors influencing hospital stay in our cohort included shorter indwelling times for intraoperative abdominal drains. Early removal or avoidance of medical devices like intra-abdominal drains may enhance postoperative mobilization and prevent atony [20]. Additionally, postoperative ICU stays were significantly shorter in the laparoscopic group, consistent with findings from the diverticulitis surgery literature [21]. In a matched-pair analysis of 110 patients, Galentin demonstrated the superiority of single-incision laparoscopy (SILS) over open surgery for sigmoid resections due to diverticulitis, showing reductions in both general hospital stays and ICU stays in the laparoscopic group.

The complication rates after surgical treatment of colovesical fistulas reported in the literature range from 6% to 49% [22]. In our cohort, the overall complication rate was 34.7%, with anastomotic leakage in 7.9%, wound infection in 21.8%, and mortality during hospitalization in 5%. These rates are slightly lower than those reported in older studies, likely reflecting advancements in surgical techniques, particularly minimally invasive approaches. Bertelson et al. reported a complication rate of approximately 37%, with an anastomotic leakage rate of 10% [23]. All of our patients underwent elective surgery, which may also account for the lower complication rates. Our findings suggest that the high proportion of laparoscopic surgeries contributed to the low overall complication rate in our cohort.

Although the difference in anastomotic leakage rates between the laparoscopic and open surgery groups did not reach statistical significance (*p* = 0.066), the observed trend is noteworthy. The absolute number of anastomotic leaks was considerably higher in the open surgery group, suggesting a potential clinical relevance that should not be overlooked. This finding aligns with existing literature indicating that minimally invasive techniques may reduce anastomotic stress and improve healing conditions due to less intraoperative tissue trauma and improved perfusion. While statistical significance was not met, the difference in leakage rates may still reflect a relevant impact of the surgical approach on patient outcomes.

The indication for surgery in colovesical or colovaginal fistulas arises from the high risks associated with these conditions and the significant impact on quality of life. Current german S3 guidelines recommend elective surgery for patients with these conditions, considering individual risk and prognosis factors [24]. For patients with limited life expectancy who may face significant risks from resective bowel surgery, alternatives like colostomy should be considered. Another option is discontinuity resection to avoid complications like anastomotic leakage. However, we advocate for biologically operable patients to undergo resective laparoscopic surgery with intestinal continuity restoration, given the limited success of other therapeutic options. A key limitation of this study is its retrospective multicenter design, which introduces the possibility of selection bias and limits the ability to draw causal inferences. In particular, more complex cases — including those requiring interprofessional surgical collaboration or presenting with extensive inflammatory changes — were more frequently treated with an open approach. Therefore, the observed differences in perioperative outcomes must be interpreted with caution. Due to the retrospective multicenter design, a systematic comparison of preoperative imaging findings with intraoperative observations was not feasible, as complete imaging datasets were not consistently available. Future prospective studies should evaluate whether specific radiologic features may predict intraoperative complexity and conversion rates. A further limitation in this study is that there is a lack of detailed anatomical documentation for the location of the fistulous tract on the bladder wall. Urological literature differentiates colovesical fistulas as supratrigonal, trigonal, or infratrigonal, for the anatomical position notably affects surgical complexity and ureteral injury risk. Many colovesical fistulas as a result of diverticulitis are in a supratrigonal location, and such a location makes them more accessible for surgical intervention. However, as previously highlighted by Melchior et al., the localization specifically of the fistula is often not systematically recorded in operative reports, as well as especially in visceral surgical settings. This underscores the need for standardized intraoperative documentation in addition to interdisciplinary collaboration in complex fistula surgery.

Our analysis reveals an association between patients’ preoperative physical status, as measured by the ASA classification, and postoperative outcomes. Specifically, both mortality and anastomotic leakage rates increased substantially with higher ASA classes. While mortality and anastomotic leakage rate was less in ASA II patients to ASA III and ASA IV. These findings emphasize the importance of thorough preoperative risk assessment and risk-adapted surgical planning. This mortality rate is within the reported range for complex fistulizing diverticulitis, particularly in patients with significant comorbidity. This corresponds to reported mortality ranges of 3–8% in series of patients undergoing surgery for fistulizing diverticulitis, especially in higher-risk populations. Notably, all ASA IV patients underwent open surgery, and the laparoscopic group included only ASA II and III patients. This suggests a deliberate selection process in clinical practice, with higher-risk patients being more frequently assigned to open surgery. This potential preoperative selection bias must be considered when interpreting outcome differences between surgical approaches. The more favorable outcomes observed in the laparoscopic group may not solely reflect advantages of the technique itself but could also be influenced by the lower-risk profile of the patients selected for this approach.

## Conclusion

Chronic sigmoid diverticulitis with the formation of colovesical or colovaginal fistulas is a surgical condition that can often be treated safely and effectively by minimally invasive resective surgery with primary restoration of bowel continuity. A laparoscopic procedure is not always possible. This depends on the complexity of the situs and the experience of the surgeon. The potential for improved postoperative outcomes supports the use of laparoscopic techniques in suitable cases. Due to the retrospective nature of this study, the results should be interpreted with caution. Further prospective research is needed to refine treatment strategies for this complex condition.

## Data Availability

No datasets were generated or analysed during the current study.
